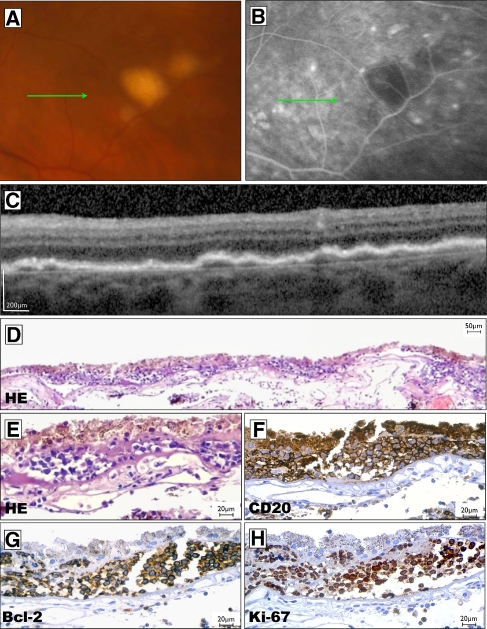# Subretinal pigment epithelial infiltrates in primary vitreoretinal lymphoma

**DOI:** 10.1007/s12348-011-0034-x

**Published:** 2011-08-06

**Authors:** Daniel Vítor Vasconcelos-Santos, Gustavo Henrique De Puy e Souza, Bernardo Bacelar de Faria, Moisés Salgado Pedrosa, André Vasconcellos Diniz, Juliana Lambert Oréfice, Rogério Alves Costa, Fernando Oréfice

**Affiliations:** 1 Department of Pathology, Universidade Federal de Minas Gerais, Belo Horizonte, Brazil; 2 Centro Brasileiro de Ciências Visuais, Belo Horizonte, Brazil; 3 Rua dos Ottoni 881/1001, Belo Horizonte, MG 30150-270 Brazil

A 67-year-old woman with chronic bilateral intraocular inflammation associated with vitreous cells and subretinal pigment epithelium (RPE) infiltrates (A–B) underwent a chorioretinal biopsy in the left eye after three negative vitreous biopsies. Sub-RPE deposits on spectral domain optical coherence tomography (C) corresponded to collections of diffuse large B-cell lymphoma cells on histopathogy (D–E) and immunohistochemistry (F–H).